# Using the ecology model to describe the impact of asthma on patterns of health care

**DOI:** 10.1186/1471-2466-5-7

**Published:** 2005-05-10

**Authors:** Barbara P Yawn, George E Fryer, Robert L Phillips, Susan M Dovey, David Lanier, Larry A Green

**Affiliations:** 1 Department of Research, Olmsted Medical Center, Rochester, MN. 55904, USA; 2 Robert Graham Policy Center, Washington, DC 20036, USA; 3 Center for Primary Care, Agency for Healthcare Research and Quality, Washington, DC, 20850, USA

## Abstract

**Background:**

Asthma changes both the volume and patterns of healthcare of affected people. Most studies of asthma health care utilization have been done in selected insured populations or in a single site such as the emergency department. Asthma is an ambulatory sensitive care condition making it important to understand the relationship between care in all sites across the health service spectrum. Asthma is also more common in people with fewer economic resources making it important to include people across all types of insurance and no insurance categories. The ecology of medical care model may provide a useful framework to describe the use of health services in people with asthma compared to those without asthma and identify subgroups with apparent gaps in care.

**Methods:**

This is a case-control study using the 1999 U.S. Medical Expenditure Panel Survey. Cases are school-aged children (6 to 17 years) and young adults (18 to 44 years) with self-reported asthma. Controls are from the same age groups who have no self-reported asthma. Descriptive analyses and risk ratios are placed within the ecology of medical care model and used to describe and compare the healthcare contact of cases and controls across multiple settings.

**Results:**

In 1999, the presence of asthma significantly increased the likelihood of an ambulatory care visit by 20 to 30% and more than doubled the likelihood of making one or more visits to the emergency department (ED). Yet, 18.8% of children and 14.5% of adults with asthma (over a million Americans) had no ambulatory care visits for asthma. About one in 20 to 35 people with asthma (5.2% of children and 3.6% of adults) were seen in the ED or hospital but had no prior or follow-up ambulatory care visits. These Americans were more likely to be uninsured, have no usual source of care and live in metropolitan areas.

**Conclusion:**

The ecology model confirmed that having asthma changes the likelihood and pattern of care for Americans. More importantly, the ecology model identified a subgroup with asthma who sought only emergent or hospital services.

## Background

Asthma is a common chronic disease affecting 5–13% of U.S. children and 3–5% of U.S. adults. [1-6] Due to its high and increasing prevalence and resulting morbidity, mortality and high cost of care, asthma is considered a priority condition by the Agency for Health Care Research and Quality (AHRQ)[7] and a major focus of Healthy People 2010 in the United States.[8]

Several studies have reported on health care utilization data for people with asthma, often focusing on emergency and hospital based care. [5,9-16] While these data are important, the designation of asthma as an ambulatory care sensitive condition demands that urgent and emergent care must be studied in the context of ambulatory visits. The ecology of medical care model is a graphical model to display the use of all types of health care by a representative group of 1000 people over a specified period of time, usually one year. The model displays the proportion of those 1000 representative people (in this case US adults and children) who have had at least one contact with the individual sites of health care services such as the emergency department, a tertiary care hospital, a clinic office or home health care services. The model does not account for the number of visits for each person but only whether or not they had any visits in that site. Each individual could be counted once in each site.[17,18]

The ecology model forces examination of the total context of health care giving the clinician, administrator or policy maker the necessary foundation for interpreting ED and hospitalization data. To date the ecology model has not been used to study the impact of a chronic disease on the interaction or sites of interaction of people and health care services. [17-19]

Using the ecology model, the 1999 Medical Expenditures Panel Survey (MEPS) provides the data needed to compare the pattern of health care contact in two population groups: people with asthma and people without any "priority" (that is, chronic, life threatening or mental health) medical conditions. The ecology model is used to demonstrate the impact of asthma on health care patterns and attempt to identify people with inappropriate patterns of care or who suffer from health care disparities.

## Methods

This is a case-control study comparing the ecology of medical care for adults and school-aged children with asthma to the ecology of care for adults and school-aged children without any known chronic, life threatening or mental health conditions. The cases and controls were selected from participants in the 1999 MEPS.

### Data Source

MEPS is sponsored by the Agency for HealthCare Quality and Research [20] and gathers information from U.S. civilian, non-institutionalized people who participated in National Health Interview Survey (NHIS).[18,19] In 1999, 23,565 persons participated in MEPS computer-assisted personal interviews.

In this study, household component records that query respondents about health care encounters in 1999 were linked with data from the MEPS Condition File describing health problems during that year. All children 6–17 and adults 18–44 years of age with self-reported asthma at the beginning of 1999 were selected as the childhood and adult asthma cases. Children less than 6 years of age were not included in this study due to the difficulty in diagnosing asthma in young children.[21] Adults 45 and older were excluded to avoid the co-mingling of people with asthma and chronic obstructive pulmonary disease. For purposes of study comparisons, adult and childhood control groups were selected that included all persons within the selected age categories (children 6–17 and adults 18–44) with no reported "priority conditions", including no asthma.

### Study Variables

Comparisons were made of the proportion of cases and controls who in 1999 had at least one contact with the health care settings assessed in MEPS including: The office of a health professional (excluding dentists and optometrists); an outpatient department; an inpatient hospital service; an emergency department (ED) or who filled a medication prescription. Personal characteristics of the cases and controls were summarized including: gender, family income, self-reported overall health, usual source of care and insurance status.

### Analytical Strategy

Descriptive analyses were used to develop the ecology boxes for cases and control subjects, analyzing adults and children separately. Analyses focused on individuals and whether they received care in each of the study settings, not the number of times seen in that setting. Relative risk ratios were calculated for visiting each of the sites, comparing patterns of use of children or adults with and without asthma. Comparisons were made within the cases (children and adults separately) stratified by the demographic and health care arrangement variables.

## Results

Asthma was reported to be present in 5% of the school-aged children (6% of boys and 4% of girls) and 3% of the adults (2% of men and 5% of women). Children and adults with asthma were more likely than those without asthma to be insured, Black, live in households at or below 300% of poverty, and report a lower overall health status. (Table 1) Adults with asthma were more likely than children with asthma to remain uninsured (12.8% versus 6.5%, P < 0.04).

**Table 1:** Demographics of children and adults with asthma using 1999 MEPS data

	**Children ages 6 through 17 years**			**Adults 18 through 45 years**		
	**Asthma %**	**No priority one conditions %**	P value*	**Asthma %**	**No priority one conditions %**	P value*
**Education**						
<HS				**18.9**	**17.3**	
HS	**NA**	**NA**	**NA**	**49.3**	**52.4**	**N**
>HS				**32.8**	**30.3**	**S**
**Rural**						
Non-MSA	**18.1**	**19.3**	**NS**	**17.1**	**16.2**	**NS**
**Gender**						**NS**
Male	**6**	**94**		**2**	**98**	
Female	**4**	**96**	**0.09**	**5**	**95**	
**Race**						
Black	**7.9**	**92.3**		**3.6**	**96.4**	**NS**
White	**4.4**	**95.6**	**<0.001**	**2.6**	**97.4**	
**Ethnicity**						
Hispanic	**3.6**	**96.4**		**1.5**	**98.6**	**<0.001**
Non-Hisp	**5.2**	**94.8**	**0.05**	**3.0**	**97.0**	
**Income level**						
Poor/Near	**30.6**	**22.13**	**NS**	**20.1**	**13.5**	
Low	**18.2**	**15.9**		**8.1**	**13.6**	**0.03**
Middle	**26.1**	**31.9**		**32.7**	**32.8**	
High	**25.1**	**30.1**		**39.2**	**40.1**	
**USC**						
Yes	**95.7**	**88.2**	**<0.01**	**81.7**	**67.2**	**<0.001**
**Perceived Health Status**						
Excellent	**22.2**	**55.4**		**11.1**	**40.2**	**<0.01**
Good/very	**62.3**	**53.0**		**70.2**	**56.2**	
Poor/Fair	**15.5**	**1.6**	**<0.01**	**18.7**	**3.6**	
**Insurance**						
Private	**65.0**	**73.4**		**75.7**	**76.6**	
Public	**28.5**	**17.3**	**0.01**	**11.5**	**5.7**	**0.01**
No	**6.5**	**9.3**		**12.8**	**17.7**	

*p-value for comparison of those with self-reported asthma compared to those with no priority conditions

People with asthma (children 95.7% and adults 81.7%) were more likely than those without asthma to have a usual source of care (Table 1). Less than 1% of adults and children with asthma listed an ED as their usual source of care. Of the one-third who reported a physician rather than a facility as their usual source of care, 66% of adults and 37% of children and teens reported that the physician was a family physician. Pediatricians were listed as the usual source of care for 56% of children and internists for 21% of adults with asthma.

As anticipated, the presence of self-reported asthma increased the likelihood of contact with the health care system in 1999 (Table 2). For example, children and teens with asthma were 1.3 times more likely to have at least one office visit during 1999 compared to the same age group without a priority condition. The comparable difference in office visits for adults was 1.6 fold.

**Table 2:** Differences in the Ecology of Medical care Associated with Self-Reported Asthma.

	**Children and teens (6–17)**			**Young adults (18 – 45)**		
**Service site**	**Self-reported Asthma %**	**No priority conditions %**	**Ratio**	**Self-reported Asthma %**	**No priority conditions %**	**Ratio**

Office	80.8	61.0	1.3	84.4	54.4	1.6
Out Pt	7.8	4.6	1.7	16.1	6.9	2.3
ED	18.5	8.2	2.2	27.2	9.4	2.9
Hospital	4.7	1.3	3.6	13.5	4.7	2.9
Hospital <24 hrs	0.6	0.2	3.2	0.6	0.2	2.5
Any prescription filled	87.3	41.2	2.1	90.5	46.9	1.9

**Figure 1:**
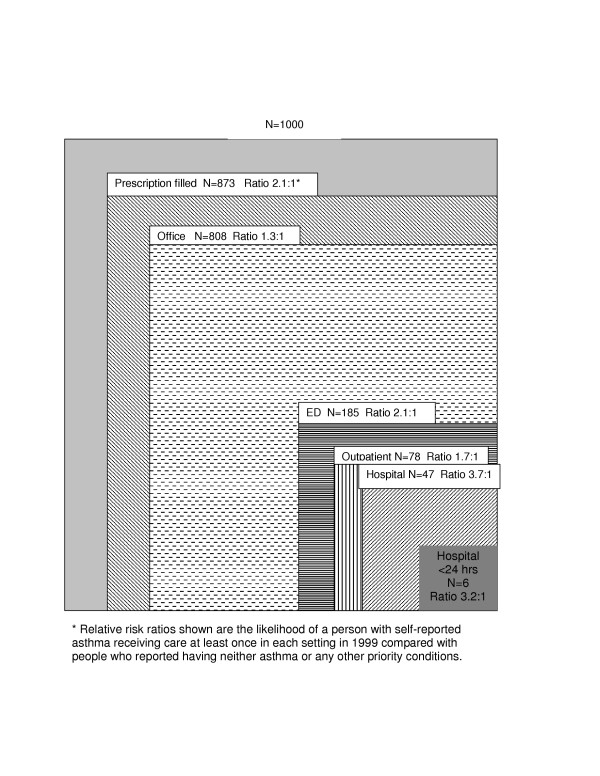
**Ecology of Asthma**. School-Aged Children (6 through 17 years)

**Figure 2:**
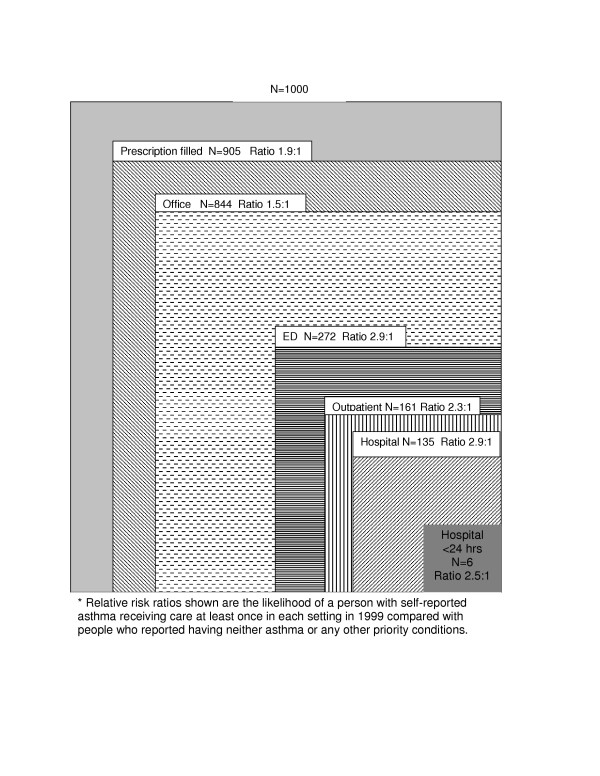
**Ecology of Asthma**. Young Adults (18 through 45 years)

Figures 1 and 2 use the ecology of medical care model to graphically illustrate the medical ecology for school-aged children and young adults and show the number of people out of 1000 who made a contact with each health care setting during 1999. The risk ratios compare those with asthma to those without chronic, life threatening or mental health conditions. Similar to previous ecology studies, [21,22] the ambulatory setting was the most common setting for care. For both children and adults with asthma, the ratio of those with at least one visit to a site was higher for the hospital (3.2 and 2.9 for children and adults respectively) and the ED (2.2 and 2.9 for children and adults, respectively) than for the office setting, suggesting that asthma has a greater impact on use of healthcare settings that provide urgent and intensive care than on ambulatory care. This relatively large difference in proportion of people contacting the ED and hospital was seen despite a significantly higher proportion of people also obtaining at least some ambulatory care. In both children and adults with asthma, filling at least one prescription was even more common than making an office visit suggesting that several people received all prescriptions from a site other than the office. This finding confirms that a group of people appears to have barriers to ambulatory care and obtains necessary drug therapy from other sites or by telephone without any office follow-up.

African American children with asthma were most likely to visit an ED or be hospitalized. They were also less likely than non-Black and Hispanic children with asthma to have any ambulatory care contact during the year. Combining office and outpatient visits, Hispanic and White (non-Hispanic) children with asthma had similar proportions of contact with ambulatory, ED and hospital care.

Some children (13.6%) and young adults (10.9%) with self reported asthma had no contact with any of the studied health care sites in 1999. Overall these people had the same average income, and racial distribution as those making at least one ambulatory visit but were more likely to be uninsured (p < 0.01) In addition, 5.2% of children and 3.6% of young adults with asthma visited the ED or were hospitalized but had no ambulatory care visits during 1999. This group was more likely to be uninsured, to have no usual source of care and to live in a metropolitan area than those with ED or hospital visits plus ambulatory visits during 1999 (p < 0.05 for each characteristic). The adults with only ED or hospital care visits also had a lower self-reported health status than adults with asthma having ambulatory visits as well as ED or hospital visits.

## Discussion

Using the ecology of medical care model to analyze MEPS data affirms that Americans with asthma are more likely to visit all healthcare sites than Americans without a chronic condition. The additional contact for the people with asthma is primarily in the ambulatory and primary care ambulatory setting. Physicians' offices appear to be an appropriate foci of care since ambulatory care visits provide the opportunity to increase the patient's and family's self-management skills, provide asthma related education and assess current asthma control. [15,16,22,23] Twenty percent of children and 16% of adults (up to 400,000 US children and 460,000 US adults) with self-reported asthma failed to make any ambulatory visit during 1999 missing these educational and monitoring opportunities. While some of these children and adults may have mistakenly reported currently active asthma, the data suggests that almost a million Americans may not be receiving adequate asthma care, if adequate care includes at least yearly visits for active asthma.

The required frequency of asthma visits when no acute exacerbations have occurred is unknown. Studies by Tirimanna and colleagues and Boom and colleagues in the Netherlands attempted to identify the prevalence of undiagnosed asthma and the required frequency of visits to minimize health care utilization.[24,25] The findings varied widely but suggested that at least once a year visits appeared beneficial.

The reasons for not making at least one ambulatory visit during a year may be enlightened by other study results from the Netherlands. In a population- based study Grunsven and colleagues found that many asthma patients were not willing to accept asthma treatment. [26] Part of the reluctance to accept therapy was a "steroid" fear [26] also reported by parents of children with asthma in the US. [27] This may explain the lack of ambulatory visits for part of this 20% of children and 16% of adults; they may quit seeking care because they do not accept the treatment recommended. The cost of co-pays to visit when the asthma is not a major problem may also discourage some less urgent asthma visits.

The lack of ambulatory visits is a special concern for a smaller group of respondents, the 5.2% of children and 3.6% of adults (approximately 130,000 U.S. children and 140,000 U.S. adults) with asthma who made an ED visit or had a hospitalization but made no ambulatory visits during the year. This group is less likely to have insurance or a usual source of care and appears to rely on urgent care sites for asthma management. The ecology model clearly highlights this group, whereas assessments based on membership in a health plan or audits of ambulatory clinics will miss this important subgroup. This is a group that may be uncommon in other countries where there all citizens are assigned a health care clinician and office.

Self-reported asthma is associated with more than twice as many people who make ambulatory visits plus visit the ED or are hospitalized compared to the control group. The reliance on ED or hospital care is most pronounced for young adults with asthma. High rates of ED visits for people with asthma have been reported previously. [9-16,21,28-32] However, few studies have attempted to anchor asthma ED visits in the context of overall pattern of care, sites visited, the presence of a usual source of care, and insurance status. This context of care is particularly important to understand since continuity ambulatory asthma management has been shown to decrease asthma-related visits to the ED and hospitalizations. [33-36] The large group with both ambulatory care and ED visits or hospitalizations may reflect people with severe and difficult to control asthma but may also identify a group without adequate ambulatory care. The ecology of medical care model is an appropriate tool to facilitate this broader view of the impact of asthma on a person's or groups' health care contacts.[17,18] The model also highlights the fact that most of the people making ED visits had a usual source of care other than the ED. This demands that we develop systems that better link ED and ambulatory care, encouraging the potential continuity of care from the ED to the office and vice versa. Several US programs are currently being studied through funding by the CDC. Results of those attempts to link the ED and the office may provide interesting and useful data.

The application of the medical ecology model to the MEPS data has limitations as well as strengths. In MEPS, asthma is self-reported but consistent with published rates of asthma prevalence. [1-3,5,41-43] In addition, the population-based data assures that the asthma experiences represent the full spectrum of disease, rather than only the moderate or severe persistent asthma that is the focus of many published studies.

The data on healthcare system encounters is also self-reported. However, the ecology model uses only the presence or absence of a health care contact. Simply remembering an encounter occurred is less subject to recall bias than remembering the timing and number of encounters.[44,45] MEPS data do not allow assessment of care appropriateness but an asthma ED or hospital visit should be followed by an ambulatory care visit. [46-49] The ecology model highlights the 3 to 5% of people with asthma who fail to access ambulatory care but use more intensive and expensive ED and hospital services.

## Conclusion

The ecology of medical care for school-aged children and young adults with self-reported asthma reveals a pattern of health care contacts that is distinctly different from those without priority conditions and identifies a group that may be the victim of health care access disparities.

## Competing interests

The author(s) declare that they have no competing interests.

## Authors' contributions

BY-initial conceptualization and design, oversaw analysis, drafted manuscript and revision, gave final approval. GF-acquired data, performed analyses, critically reviewed manuscript and revisions, gave final approval. SD-same as RP. RP participated in design and critical review of manuscript and gave final approval. DL- same as RP. LAG-same as RP & DL

## Pre-publication history

The pre-publication history for this paper can be accessed here:


